# Endoplasmic Reticulum-Localized PURINE PERMEASE1 Regulates Plant Height and Grain Weight by Modulating Cytokinin Distribution in Rice

**DOI:** 10.3389/fpls.2020.618560

**Published:** 2020-12-22

**Authors:** Yunhua Xiao, Junwen Zhang, Guiyuan Yu, Xuedan Lu, Wentao Mei, Huabing Deng, Guilian Zhang, Guihua Chen, Chengcai Chu, Hongning Tong, Wenbang Tang

**Affiliations:** ^1^Southern Regional Collaborative Innovation Center for Grain and Oil Crops in China, College of Agriculture, Hunan Agricultural University, Changsha, China; ^2^State Key Laboratory of Plant Genomics, Institute of Genetics and Developmental Biology, Innovative Academy of Seed Design, Chinese Academy of Sciences, Beijing, China; ^3^National Key Facility for Crop Gene Resources and Genetic Improvement, Institute of Crop Sciences, Chinese Academy of Agricultural Sciences, Beijing, China

**Keywords:** purine permease, cytokinin, plant height, grain weight, rice (*Oryza sativa* L.)

## Abstract

Cytokinins (CKs) are a class of phytohormones playing essential roles in various biological processes. However, the mechanisms underlying CK transport as well as its function in plant growth and development are far from being fully elucidated. Here, we characterize the function of PURINE PERMEASE1 (OsPUP1) in rice (*Oryza sativa* L.). *OsPUP1* was predominantly expressed in the root, particularly in vascular cells, and CK treatment can induce its expression. Subcellular localization analysis showed that OsPUP1 was predominantly localized to the endoplasmic reticulum (ER). Overexpression of *OsPUP1* resulted in growth defect of various aerial tissues, including decreased leaf length, plant height, grain weight, panicle length, and grain number. Hormone profiling revealed that the CK content was decreased in the shoot of *OsPUP1*-overexpressing seedling, but increased in the root, compared with the wild type. The CK content in the panicle was also decreased. Quantitative reverse transcription-PCR (qRT-PCR) analysis using several CK type-A *response regulators* (*OsRRs*) as the marker genes suggested that the CK response in the shoot of *OsPUP1*-overexpressing seedling is decreased compared to the wild type when CKs are applied to the root. Genetic analysis revealed that BG3/OsPUP4, a putative plasma membrane-localized CK transporter, overcomes the function of OsPUP1. We hypothesize that OsPUP1 might be involved in importing CKs into ER to unload CKs from the vascular tissues by cell-to-cell transport.

## Introduction

Cytokinins are a class of phytohormones playing important roles in various biological processes including cell division and differentiation, shoot/root balance, nutrient relocation, seed number as well as stress responses (Sakakibara, 2006). In plants, CKs exist naturally either in free-base forms, including *N*^6^-(Δ^2^-isopentenyl)-adenine (iP), *trans-*zeatin (tZ), *cis-*zeatin (cZ), and dihydrozeatin (DHZ), or in conjugated forms, although the ratio varies with plant species (Sakakibara, 2006; Osugi and Sakakibara, 2015). A series of synthetic enzymes, including isopentenyltransferases (IPTs), CK-specific cytochrome P450 (CYP735As), and LONELY GUY/LOG LIKE phosphoribohydrolases (LOG/LOGLs), have been identified, while uridine diphosphate glucosyltransferases (UGTs) and CK oxidase/dehydrogenases (CKXs) are involved in the CK inactivation and degradation (Sakakibara, 2006; Kurakawa et al., 2007). IPTs preferably utilize adenosine triphosphate (ATP) or adenosine diphosphate (ADP) as isoprenoid acceptors to synthesize isopentenyladenine riboside 5′-triphosphate (iPRTP) and isopentenyladenine riboside 5′-diphosphate (iPRDP), respectively (Kakimoto, 2001); CYP735As convert iP nucleotides into the corresponding tZ nucleotides (Takei et al., 2004); LOG/LOGLs catalyze the transition from inactive CK derivatives to bioactive CK nucleobases (Kurakawa et al., 2007; Kuroha et al., 2009); UGTs deactivate CK nucleobases by conjugation at *O*- and *N*- position with a sugar moiety, mostly glucose (Martin et al., 1999a, b; Šmehilová et al., 2016). CKXs catabolize CKs to adenine or adenosine (Galuszka et al., 2001).

The signal pathway of CK involves a His-Asp phosphorelay system from receptor histidine kinases (HKs) to histidine-containing phosphotransfer proteins (HPTs), then to the transcriptional factor type-B response regulators (RRs) (Werner and Schmülling, 2009; Hwang et al., 2012; Kieber and Schaller, 2018). Hybrid HKs sense CKs via the cyclases/histidine kinases associated sensory extracellular (CHASE) domain for CK-binding, which reside both in plasma membrane (PM) and ER, and have been suggested to mainly happen in ER lumen (Caesar et al., 2011; Lomin et al., 2011, 2018; Wulfetange et al., 2011; Hwang et al., 2012; Ding et al., 2017; Romanov et al., 2018; Kubiasová et al., 2020). Type-B RRs contain DNA-binding domain and mediate CK-dependent transcriptional activation (Sakai et al., 2000, 2001; Hwang and Sheen, 2001; Hutchison et al., 2006). Type-B RRs regulate the expression of target genes in response to the hormone (Sakai et al., 2000, 2001). Among the target genes, type-A RRs are induced by CK and play negative roles through competing with type-B RRs for phosphoryl group (Werner and Schmülling, 2009; Hwang et al., 2012; Kieber and Schaller, 2018).

Cytokinins regulate various agronomic traits, such as grain number, grain size, and plant height. Loss-of-function of *LOG* decreases shoot apical meristem and reduces grain number (Kurakawa et al., 2007). Knockout of *CYP735A4* decreases plant height (Gao et al., 2019). Decreased expression of *OsCKX2/Gn1a* increases the grain number (Ashikari et al., 2005; Li et al., 2013). *OsCKX2* also negatively regulates grain weight (Yeh et al., 2015). The knockout mutants of *OsCKX11* display delayed leaf senescence and increased grain number (Zhang W. et al., 2020). Overexpression of another CK oxidase/dehydrogenase gene *OsCKX4* significantly decreases grain number, grain weight, and plant height (Gao et al., 2014). *TaCKX6-D1*, a wheat ortholog of rice *OsCKX2*, has been shown to be significantly associated with grain weight, and haplotype of the gene is associated with higher grain weight (Zhang L. et al., 2012). Knockdown of *TaCKX2.4* increases grain numbers per spike (Li et al., 2018). However, it has been suggested that *TaCKX2.1* and *TaCKX2.2* expressions are positively correlated with grain number per spike (Zhang J. et al., 2011).

Cytokinins function not only as local paracrine signal, but also as long-distance signal through translocating in vascular tissues (Sakakibara, 2006; Hirose et al., 2008; Osugi and Sakakibara, 2015; Liu et al., 2019). Trace experiments with the help of isotope-labeled CKs have demonstrated the movement of CK among tissues *in planta* (Bishopp et al., 2011; Kiba et al., 2013; Sasaki et al., 2014; Zhang K. et al., 2014). Due to the tissue-specific expression pattern of CK biosynthetic genes such as *CYP735As* which are mainly expressed in the roots for synthesis of tZ-type CKs, CK species are unevenly produced in different tissues (Takei et al., 2004; Hirose et al., 2008). tZ-type CKs are mainly distributed in xylem sap, while iP-type CKs mainly present in the phloem sap (Hirose et al., 2008). Moreover, it has been demonstrated that the shoot-derived and root-derived CKs could have specific function in regulating plant growth and development (Matsumoto-Kitano et al., 2008; Kiba et al., 2013; Sasaki et al., 2014). The *Arabidopsis atipt1;3;5;7* quadruple mutant with reduced CK content does not form cambium and displays reduced thickness of the stem and root (Matsumoto-Kitano et al., 2008). Reciprocal grafting the shoot and root of the quadruple mutant and the wild-type plant recover the growth-deficient phenotypes of the mutant (Matsumoto-Kitano et al., 2008). The *Arabidopsis cyp735a1 cyp735a2* double mutant with severely reduced tZ-type CK content but unchanged total CK quantity has retardation of the shoot growth, which can be recovered to the wild-type phenotype by applying exogenous tZ but not iP (Kiba et al., 2013). The shoot phenotype can also be complemented with the recovery of tZ-type CK content by grafting the shoot of the double mutant onto the wild-type stock (Kiba et al., 2013).

There are at least four types of proteins reported to be involved in CK traffic and translocation. One type is ATP-binding cassette (ABC) transporter subfamily. *AtABCG14* is expressed in cells of vascular tissues and localized to the plasma membrane and it functions as an efflux transporter for loading CK into xylem, and plays a crucial role in the long distance transport of root-derived CKs (Ko et al., 2014; Zhang K. et al., 2014). A rice homolog, *OsABCG18*, has been shown to play a similar role (Zhao et al., 2019). Loss-of-function of either *AtABCG14* in *Arabidopsis* or *OsABCG18* in rice leads to the retention of tZ-type CKs in the roots, resulting in reduced growth of the shoots (Ko et al., 2014; Zhang K. et al., 2014; Zhao et al., 2019). Another type is equilibrative nucleoside transporter (ENT) family, which has been suggested to selectively translocate CK nucleosides (Hirose et al., 2005, 2008). *OsENT2* is expressed in the scutellum of germinating seeds and the vascular tissues of germinated seedlings, and predominantly expressed in the roots in mature plants (Hirose et al., 2005). It has been suggested that OsENT2 participates in retrieving endosperm-derived nucleosides through the germinating embryo and in the long-distance transport of nucleosides in growing plants (Hirose et al., 2005). Three homologs in *Arabidopsis*, AtENT3, AtENT6, and AtENT8, are also suggested to be involved in transporting CK nucleoside (Sun et al., 2005; Hirose et al., 2008). Very recently, AZG2, a member of AZA-GUANINE RESISTANT (AZG) purine transporter family, is reported to have the ability to transports purines and CK with high affinity (Tessi et al., 2020). The forth type is purine permease (PUP) family. Three genes, *AtPUP1*, *AtPUP2*, and *AtPUP14*, are supposed to mediate CK nucleobase uptake in *Arabidopsis* (Bürkle et al., 2003; Zürcher et al., 2016). *AtPUP1* is expressed in the epithem of hydatodes and the stigma surface of silique, and localized to the plasma membrane, whereas *AtPUP2* is expressed in the phloem of leaves (Bürkle et al., 2003; Szydlowski et al., 2013). AtPUP14 is also localized to the plasma membrane, and has the ability to import CK nucleobase into cell (Zürcher et al., 2016). AtPUP14 is proposed to diminish the spatiotemporal active CK sink in the apoplast for perception by plasma membrane-localized CK receptor (Zürcher et al., 2016). In rice, there are 12 PUP family members (Qi and Xiong, 2013). OsPUP4 and OsPUP7 are localized to the plasma membrane and endoplasmic reticulum (ER), respectively, though they are both expressed in vascular tissues (Qi and Xiong, 2013; Xiao et al., 2019). OsPUP4 and OsPUP7 are assumed to be involved in long-distance transport and local allocation of CK in a cell-to-cell way (Xiao et al., 2019).

In this study, we identified another PUP homolog OsPUP1. The gene was expressed highly in the root, predominantly in vascular cells, and the protein was predominantly localized to ER. Overexpression of *OsPUP1* led to altered distribution of CKs, and resulted in growth defect in the shoot. Further analyses suggested that the CK response in *OsPUP1*-overexpressing seedling plant is altered. We hypothesize that OsPUP1 might be involved in importing CKs into ER to mediate CK transport and CK response.

## Materials and Methods

### Plant Materials and Growth Conditions

The *Japonica* cultivar Zhonghua11 was used as the wild type in this study. For the analysis at the reproductive stage, rice plants were grown in the field under natural condition. For seedling analysis, rice plants were grown hydroponically in a growth chamber at 28°C with a 12-h-day/12-h-night cycle, light intensity of 30000 lux, and humidity of 70%. Modified Kimura B (pH 5.8) solution (Ma et al., 2001) was supplied as nutrient medium containing the following macronutrients (mM): (NH_4_)_2_SO_4_ (0.36), MgSO_4_.7H_2_O (0.54), KNO_3_ (0.18), Ca(NO_3_)_2_ (0.36), K_2_SO_4_ (0.09), KH_2_PO_4_ (0.18), and Na_2_SiO_3_.9H_2_O (1.6); and micronutrients (μM): MnCl_2_.4H_2_O (9.14), H_3_BO_3_ (46.2), H_2_MoO_4_ (0.56), ZnSO_4_.7H_2_O (0.76), CuSO_4_.5H_2_O (0.32), and Fe(II)-EDTA (20).

### Vector Construction and Plant Transformation

The full-length coding sequence of *OsPUP1* was cloned into pCAMBIA2300-Actin and pCAMBIA2300-35S:GFP to generate the constructs for overexpression and protein subcellular localization analysis, respectively. The 2,091 bp promoter sequence upstream the start codon of *OsPUP1* was cloned into pCAMBIA2391Z to generate the construct for GUS staining analysis. Sequences were cloned into vectors by recombination fusion strategy. To create knockout mutants, *OsPUP1* was edited by targeting 5′-GTCGTGCTTCGTGTACGCGCTGG-3′ in the coding sequence as described previously (Lu et al., 2017). The transgenic plants were produced using Zhonghua11 as the receptor by *Agrobacterium tumefaciens*-mediated transformation following the previously described method (Liu et al., 2007). T_0_ and T_1_ lines of *pOsPUP1:GUS* transgenic plants, and T_3_ and higher lines of *OsPUP1-*overexpressing and knockout homozygous plants were used for analyses.

### Total RNA Isolation and qRT-PCR Analysis

Total RNA was isolated using TRIzol (Code No. 15596026, Invitrogen). The cDNA was synthesized using a kit named “PrimeScript^TM^ RT reagent Kit with gDNA Eraser” (Code No. RR047A, TaKaRa) following the product instructions. qRT-PCR using SYBR Green PCR mix (Code No. RR820A, TaKaRa) was performed on a real-time PCR detection system (Bio-Rad CFX96) according to the manufacturer’s instructions. The rice *Ubiquitin2* gene was used as an internal reference for all analyses. The primers used for qRT-PCR are listed in Supplementary Table 2.

### GUS Staining

Root from plants at the seedling stage and other tissues from plants at the reproductive stage were sampled for GUS staining according to a previously described method (Jefferson, 1989). The stained tissues were observed and the images were taken using a stereomicroscope (Olympus SZX16) with a digital camera (Canon EOS 600D).

### Hormone Treatment

For responsive analyses of *OsPUP1* to CK as well as other phytohormones, the roots of 8-day-old wild-type seedlings were treated with iP, tZ, or cZ at 1 μM concentrations for 2 h, or treated with other phytohormones, including brassinolide (BL), gibberellin (GA_3_), abscisic acid (ABA), 1-aminocyclopropane-1-carboxylic acid (ACC), indole-3-acetic acid (IAA), and jasmonic acid (JA), at 10 μM concentrations for 4 h. The materials for the analyses were used as the same as in our previous work (Xiao et al., 2019). For CK transport analysis, the roots of 10-day-old wild-type seedlings and *OsPUP1*-overexpressing seedlings were treated using iP, tZ, or cZ at 0.01 μM concentrations for 4 h. After treatments, the shoots and roots of the plants were separately harvested for expression analyses of *OsPUP1*, *OsRR1*, *OsRR2*, and *OsRR4*.

### Measurement of CKs

Shoots and roots of 10-day-old rice seedlings grown in a growth chamber and 18–20 cm length panicles of plants grown in the field under natural condition were harvested and used for measurement of CKs as described previously (Cai et al., 2014).

### Subcellular Localization Analysis of OsPUP1

pCAMBIA2300-35S:GFP-OsPUP1 was transformed alone or co-transformed with endoplasmic reticulum-red fluorescent protein (ER-RFP) into rice protoplasts using a previously described method (Zhang Y. et al., 2011). The same vector was introduced into *Nicotiana benthamiana* leaves by *Agrobacterium tumefaciens*-mediated transformation following the method described previously (Sparkes et al., 2006). After incubating for 18 h in rice protoplasts and 48 h in tobacco leaves, fluorescent signals were detected using a confocal laser scanning microscopy (Leica TCS SP5).

### Phylogenetic Analysis

*PUP* genes in *Arabidopsis*, coffee, and rice are numbered according to previous studies (Qi and Xiong, 2013; Zürcher et al., 2016; Kakegawa et al., 2019). Gene information referred to websites for *Arabidopsis*^1^, coffee^2^, and rice^3^. Protein sequences were used to construct the phylogenetic tree by software MEGA X (Kumar et al., 2018) using the Maximum Likelihood method based on the JTT matrix-based model (Jones et al., 1992). The tree was drawn to scale, with branch lengths measured in the number of substitutions per site.

### Accession Numbers

Sequence data from this article can be found in the Rice Genome Annotation Project (see text footnote 3) under the following accession numbers: *OsPUP1* (LOC_Os03g08880), *BG3/OsPUP4* (LOC_Os01g48800), *OsRR1* (LOC_Os04g36070), *OsRR2* (LOC_Os02g35180), *OsRR4* (LOC_Os01g72330), and *Ubiqutin2* (LOC_Os02g06640).

## Results

### Molecular Characteristics of OsPUP1

**FIGURE 1:**
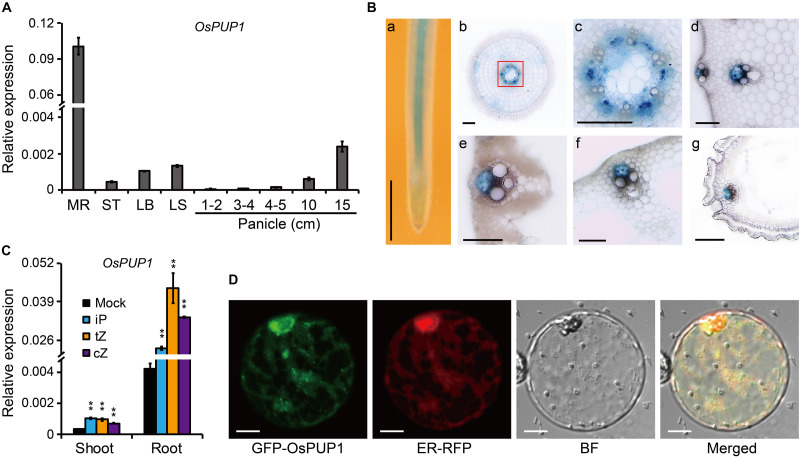
Molecular characteristics of OsPUP1. **(A)** Expression pattern of *OsPUP1* in various tissues of the wild-type plant. MR, mature root; ST, stem; LB, leaf blade; LS, leaf sheath. *Ubiquitin2* gene was used as the internal reference. *n* = 3, bar = SD. **(B)** GUS staining analyses of the various tissues of the *pOsPUP1:GUS* transgenic plants. **(a)** young root; **(b)** cross section of young root; **(c)** amplification of the red-framed regions in **(b)** to better show the signals in phloem; cross section of culm **(d)**, leaf blade **(e)**, leaf sheath **(f)**, and grain husk **(g)**. Scale bar: 1 mm in **(a)**, 50 μm in **(b–g)**. **(C)** Inductive effect of different CK nucleobases on *OsPUP1* expression in the shoots and roots of 8-day-old wild-type seedlings, of which roots were treated by iP, tZ, and cZ at 1 μM concentrations for 2 h. *Ubiquitin2* gene was used as the internal reference. *n* = 3, bar = SD, **P < 0.01 in Student’s *t*-test. **(D)** Subcellular localization of OsPUP1 in rice protoplast. ER, endoplasmic reticulum; BF, bright field. Scale bar, 10 μm.

We previously identified two OsPUPs, BG3/OsPUP4, and OsPUP7, being involved in long-distance transport of CK (Xiao et al., 2019). OsPUP1 is a close homolog of OsPUP4 and OsPUP7. In addition, it has been shown that both OsPUP1 and OsPUP4 have increased expression in *OsPUP7*-overexpressing plant (Qi and Xiong, 2013). We thus selected OsPUP1 for further analysis in order to explore its potential role in CK transport. We firstly evaluated the expression pattern of *OsPUP1* in different tissues of the wild-type plant by qRT-PCR. The results showed that *OsPUP1* was evidently expressed in all tissues tested, including mature root, stem, leaf blade, leaf sheath, and panicles with different length (Figure 1A). However, the expression level was much higher in the root than those of other tissues (Figure 1A). In reproductive tissues, *OsPUP1* expression was gradually increased along with the panicle development (Figure 1A). This expression pattern was somewhat similar with those of *OsPUP4* and *OsPUP7* (Xiao et al., 2019), indicating that OsPUP1 could also play a role in panicle growth and development.

To further dissect the expression pattern of *OsPUP1*, we constructed a plasmid with GUS driven by the promoter of *OsPUP1* and introduced it into the wild-type plant. Histochemical staining of various tissues, including root, stem, leaf blade, leaf sheath, young panicle, and husk, showed that *OsPUP1* was predominantly expressed in vascular tissues, and turned to be specific in phloem (Figure 1B). The expression was also detected in other cells such as parenchymal cells, but to a much lesser extent (Figure 1B).

To test whether *OsPUP1* is responsive to CK, we analyzed the *OsPUP1* expression under CK treatment. iP, tZ, and cZ are three type of active CKs that can be endogenously synthesized in rice. When the roots of the wild-type seedling were treated with these different CKs, respectively, *OsPUP1* was always significantly induced in both shoots and roots (Figure 1C), suggesting that CKs can positively regulate the expression of *OsPUP1*. We further examined the expression of *OsPUP1* under other phytohormone treatments. Interestingly, *OsPUP1* could also be induced by brassinolide (BL), gibberellin (GA), 1-aminocyclopropane-1-carboxylic acid (ACC), and jasmonic acid (JA), but was suppressed by abscisic acid (ABA) and indole-3-acetic acid (IAA) (Supplementary Figure 1). Thus, it appears that OsPUP1 as a potential CK transporter is involved in response to various phytohormones.

Subcellular localization of a protein is important for its function, and OsPUP4 has been shown to be localized on plasma membrane for CK transport (Xiao et al., 2019). We thus tagged OsPUP1 with a green fluorescent protein (GFP) tag at the N-terminus of the protein and then introduced the corresponding vector into either rice protoplast or tobacco epidermal cells for analysis. Observation with a confocal laser scanning microscopy showed that OsPUP1 was apparently not localized to plasma membrane in protoplast (Supplementary Figure 2), but appeared to be localized to the endoplasmic reticulum (ER), as the nuclei were surrounded by the fluorescence signal (Supplementary Figure 3), which is thought to be a typical characteristic of ER localization (Sparkes et al., 2006). To confirm this result, we co-expressed the fusion protein with an ER marker (ER-RFP) in rice protoplasts, and found the fluorescence signals of the two fusion proteins are highly overlapped, demonstrating that OsPUP1 was predominantly localized to ER.

### Overexpression of *OsPUP1* Suppresses Plant Height, Grain Weight and Grain Number

**FIGURE 2:**
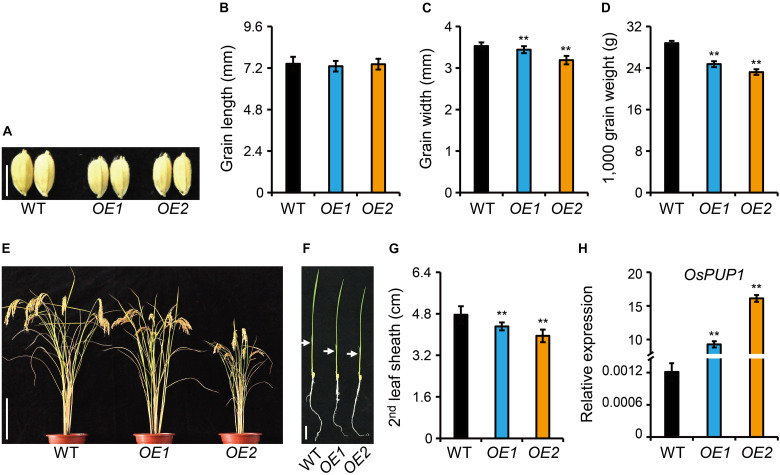
Phenotype analyses of *OsPUP1*-overexpressing plants. **(A)** Comparison of the grain morphology of the wild-type plant (WT) and two representative *OsPUP1*-overexpressing plants, designated as *OE1* and *OE2* for short. Scale bar, 5 mm. **(B–D)** Statistical data of the grain length **(B)**, grain width **(C)**, and grain weight **(D)** in **(A)**. *n* = 20 in **(B,C)**, *n* = 3 in **(D)**, bar = SD, **P < 0.01 in Student’s *t*-test. **(E,F)** Gross morphology of 140-day-old plants at the reproductive stage **(E)** and 7-day-old plants at the seedling stage **(F)**. Scale bar: 20 cm in **(E)** and 2 cm in **(F)**. White arrowheads in **(F)** mark the second leaves. **(G)** Statistical data of the second leaf sheath length in **(F)**. *n* = 9, bar = SD, **P < 0.01 in Student’s *t*-test. **(H)** Relative expression of *OsPUP1* in shoots of 10-day-old *OsPUP1*-overexpressing seedlings tested by qRT-PCR, compared with WT. *Ubiquitin2* gene was used as the internal reference. *n* = 3, bar = SD, **P < 0.01 in Student’s *t*-test.

To study the function of OsPUP1 in regulating growth and development in rice, we overexpressed *OsPUP1* under the control of *ACTIN1* promoter in the wild-type plants, and obtained a number of independent transgenic plants. Compared with the wild-type plant, homozygous *OsPUP1*-overexpressing plants (designated as *OE* for short) exhibited remarkably reduced growth of various tissues. In detail, the grain size and grain weight were significantly decreased, which could be mainly attributed to the reduction of grain width (Figures 2A–D). The plant height was also decreased both at the reproductive stage and at the seedling stage (Figures 2E,F and Supplementary Figure 4A). In addition, the tiller number of the transgenic plants was slightly less than the wild type (Supplementary Figure 4B). Both the leaf blade and leaf sheath were shorter than the wild type (Figure 2G and Supplementary Figure 4C). Moreover, the panicle length, the primary and secondary branches were all decreased, which finally resulted in a decreased grain number (Supplementary Figure 5). The severity of the above-mentioned phenotypes were well consistent with the expression level of *OsPUP1* (Figure 2H).

We also generated knockout mutants of *OsPUP1* using CRISPR/Cas9 gene-editing technology. Two independent homozygous lines, both containing frameshift mutations with 1 bp insertion in the coding region and thus should be knockout alleles, were selected for phenotypic analysis (Supplementary Figure 6A). The mutation seems to have no effect on gene transcription since the expression of *OsPUP1* was not changed in both mutants (Supplementary Figure 6B). However, no clear phenotypic difference was observed compared with the wild-type plant, suggesting the existence of functional redundancy among PUP members.

Since *ospup1* mutant is phenotype-silent, we next focused on the analysis of the overexpressing plants for dissection of potential functions of OsPUP1. Considering that *OE1* showed a weak phenotype and even had no statistically significant difference in some terms compared with the wild type (Supplementary Figures 4, 5), whereas *OE2* presented a very typical and marked phenotype, we majorly used *OE2* as a representative line for the following analyses.

### *OsPUP1-*Overexpressing Plants Have Reduced CK Levels in Shoot and Panicle

**FIGURE 3:**
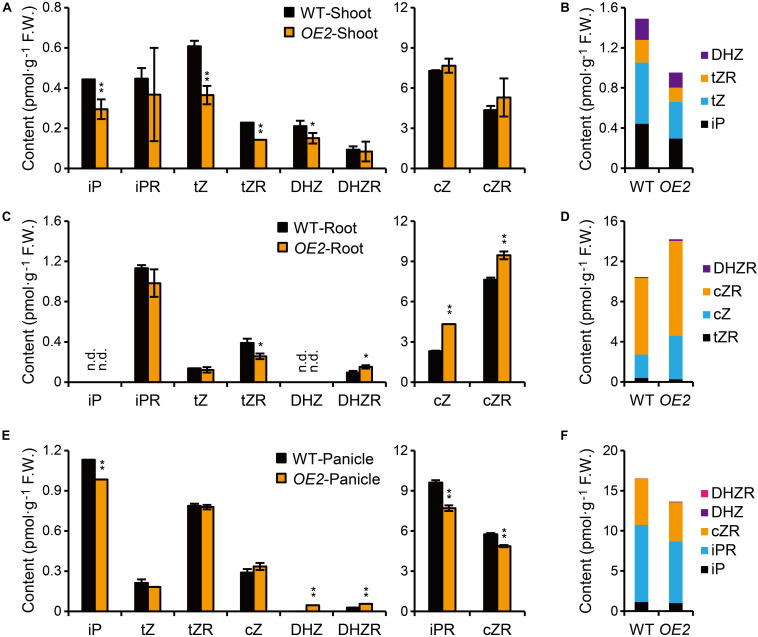
Quantification of CKs in *OsPUP1*-overexpressing plants. **(A,C,E)** Quantification of CKs in shoots **(A)** and roots **(C)** of 10-day-old seedlings and panicles **(E)** with 18–20 cm length of the wild-type (WT) and *OsPUP1*-overexpressing plants (*OE2*). See Supplementary Table 1 for individual values. Different CK forms were grouped according to the content. *n* = 3, bar = SD. n.d., not detected. *P < 0.05 and **P < 0.01 in Student’s *t*-test. Asterisks indicate statistically significant difference of *OE2*, compared with WT. **(B,D,F)** Total amounts of the biologically active forms of CKs with statistical significance in shoots **(B)**, roots **(D)**, and panicles **(F)**. FW, fresh weight.

Given the potential role of PUPs in CK transport, we asked whether the marked phenotypic changes of *OsPUP1*-overexpressing plants are associated with alteration of CK contents. To this end, we directly quantified various CK forms in both the shoot and the root of *OsPUP1*-overexpressing seedlings, respectively. CK nucleobases are thought to be solely active CK forms (Sakakibara, 2006; Hothorn et al., 2011; Lomin et al., 2015), and CK nucleosides can be easily transformed *in vivo* to CK nucleobases (Yonekura-Sakakibara et al., 2004; Hwang et al., 2012). Compared to the wild-type plants, the content of iP, tZ, and DHZ, three kinds of CK nucleobases, and the nucleoside form of tZ (tZR) were reduced, but cZ and other nucleoside forms tested were not markedly changed in the shoot of *OsPUP1*-overexpressing plants (Figure 3A). However, cZ, cZR, and DHZR were increased, tZR was decreased, and other biologically active forms were not markedly changed in the root (Figure 3C). Interestingly, the most abundant inactive form *cis-*zeatin *O*-glucoside (cZOG) increased in both shoots and roots (Supplementary Table 1). Nevertheless, the significant reduction of the total CK nucleobases and nucleosides in the shoot might explain the dwarfism phenotype of *OsPUP1*-overexpressing seedling plant (Figure 3B).

It has been shown that CKs also promote grain size and grain number in rice (Ashikari et al., 2005; Gao et al., 2014; Xiao et al., 2019; Yin et al., 2020). To test whether the decrease of grain size and grain number in *OsPUP1*-overexpressing plants are associated with the alteration of CK content, we further quantified the CK content in the panicles (Figures 3E,F and Supplementary Table 1). Compared with the wild-type plants, iP, iPR, and cZR were all significantly decreased in the transgenic plants, but DHZ and DHZR were slightly increased, while other biologically active forms were not significantly changed (Figure 3E). Thus, the decreased grain weight of *OsPUP1*-overexpressing plants might be caused by the reduction of the total content of CK nucleobases and nucleosides in the panicles (Figure 3F).

### Shoot of *OsPUP1*-Overexpressing Plants Exhibits Decreased Response to CK Application in Root

**FIGURE 4:**
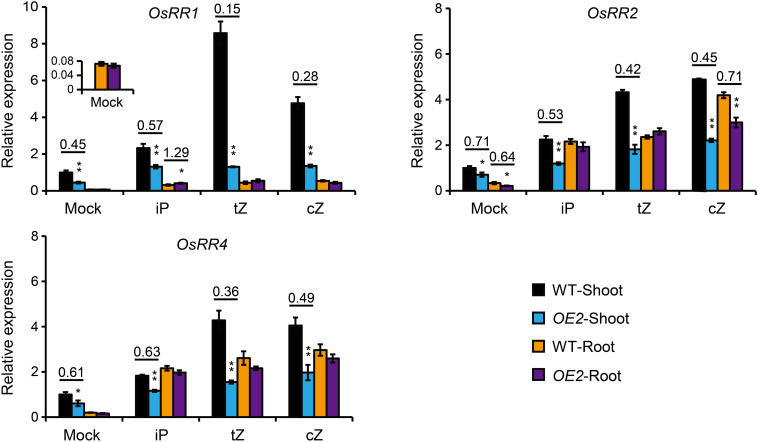
Expression analyses of *OsRR* genes in the root and shoot of the seedlings treated by different forms of CKs in the root. The roots of 10-day-old seedlings of *OsPUP1*-overexpressing plant (*OE2*) and the wild type (WT) were treated by mock solution, or different CK nucleobases at 0.01 μM concentrations for 4 h, and then the shoot and root tissues were separately collected for gene expression analyses. *Ubiquitin2* gene was used as the internal reference. Genes and tissues are marked in each panel. *n* = 3, bar = SD. *P < 0.05 and **P < 0.01 in Student’s *t*-test. The values of WT shoots without CK treatment (Mock) were set to 1, and other values were the relative values compared to them. Asterisks indicate statistically significant difference of *OE2*, compared with WT. The pairs with statistically significant difference were calculated for the ratios of the relative expression of *OE2* to WT.

The levels of tZ and tZR, two CK forms mainly synthesized in the root (Takei et al., 2004; Xiao et al., 2019), were decreased in the shoot of *OsPUP1*-overexpressing seedlings, and the significant increase of cZ and cZR in the root did not lead to the accordingly increase of the two forms in the shoot (Figure 3A). Given that OsPUP1 could be a CK transporter, we hypothesized that the root-to-shoot transport of CK was impaired in the transgenic plants. To test this possibility, we treated the roots of both *OsPUP1*-overexpressing plants and the wild-type with three kinds of CK nucleobases, including iP, tZ, and cZ, at 0.01 μM concentrations for 4 h, and then tried to compare the CK response in the shoots. If the root-to-shoot transport of CK were altered in the plant, the hormone response in the shoot should be accordingly altered in response to the CK treatment in the root. Three A-type *OsRR* genes, *OsRR1*, *OsRR2*, and *OsRR4*, which are sensitively induced by CK (Kudo et al., 2012; Tsai et al., 2012; Xiao et al., 2019), were used as the marker genes to analyze the CK response in the shoot and root, respectively, and thus to indicate the activity of CK signal transduction. Without treatment, the expression levels of these *OsRR* genes were decreased in the shoot of *OsPUP1*-overexpressing plants, consistent with the decreased CK level, but unchanged in the root, compared with the wild-type (Figure 4). Upon CK treatment in the roots, the expression levels were significantly induced in the roots of both *OsPUP1*-overexpressing plants and the wild type (Figure 4). In the shoots, the expression levels of the three genes were induced in both *OE2* and the wild type under either tZ or cZ treatment, however, the induction extents are much lower in *OE2* than those in the wild type (Figure 4). For iP treatment, similar tendency was also observed, although the extent was much lower compared to those in tZ and cZ treatment (Figure 4). Taken together, these results strongly suggested that the long-distance transport of CK from root to shoot is reduced in *OsPUP1*-overexpressing plants.

### BG3/OsPUP4 Overcomes OsPUP1 Function

**FIGURE 5:**
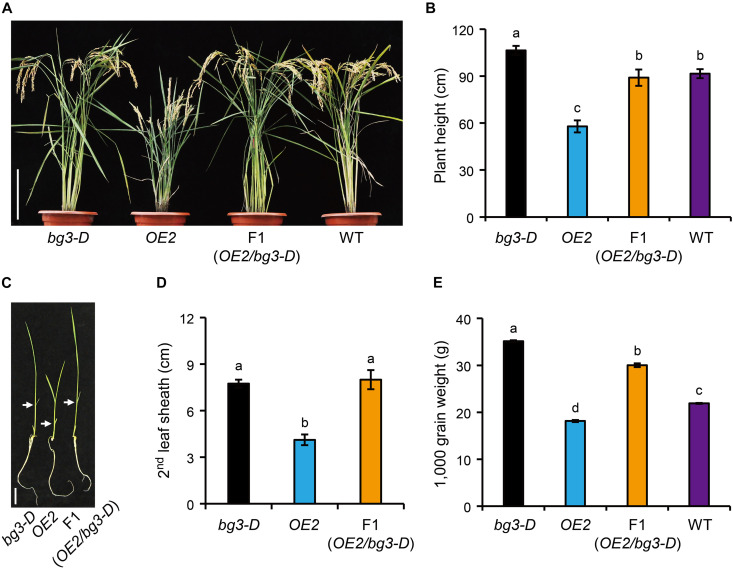
Analysis of the relationship between BG3/OsPUP4 and OsPUP1. **(A,C)** Comparison of the gross morphology of the *bg3-D* mutant, *OsPUP1*-overexpressing plant, and their F_1_ progeny, in which both of *BG3/OsPUP4* and *OsPUP1* were overexpressed, at 128-day-old reproductive stage **(A)** and 12-day-old seedling stage **(C)**. White arrowheads in **(C)** mark the second leaves. Scale bar: 20 cm in **(A)**, 4 cm in **(B)**. **(B,D,E)** Statistical data of plant height in **(A)**, the second leaf sheath length **(D)** in **(C)**, and grain weight **(E)**. *n* = 8 in **(B)**, *n* = 5 in **(D)**, *n* = 3 in **(E)**, bar = SD. Different letters above the columns indicate statistically significant differences between groups (*t* Test LSD, *P* < 0.05).

Phylogenetic analysis of PUPs involving three different plant species, including *Arabidopsis*, coffee, and rice, showed that OsPUP1 is close to BG3/OsPUP4 as well as OsPUP7 and OsPUP8 in rice (Supplementary Figure 7). However, our results clearly revealed that overexpression of *BG3/OsPUP4* and *OsPUP1* led to significantly different or even opposite phenotypes (Xiao et al., 2019). To study the relationship between the two genes, we crossed *bg3-D* mutant, in which *BG3/OsPUP4* expression is activated (Xiao et al., 2019), with *OsPUP1*-overexpressing plant. At the reproductive stage, the F_1_ plants with both *BG3/OsPUP4* and *OsPUP1* overexpressed exhibited a similar plant height as the wild type (Figures 5A,B and Supplementary Figure 8). Similar result was obtained at the seedling stage, as the F_1_ plants showed a plant height like *bg3-D* (Figures 5C,D), which is taller than the wild type due to longer leaves (Xiao et al., 2019), suggesting that activation of *BG3* may mask the effect of *OsPUP1*-overexpression. In addition, the F_1_ plants also showed increased grain weight (Figure 5E). Taken together, these results strongly suggested that, although the two genes function antagonistically in regulating plant growth and development, BG3/OsPUP4 can somehow overcome the role of OsPUP1.

## Discussion

**FIGURE 6:**
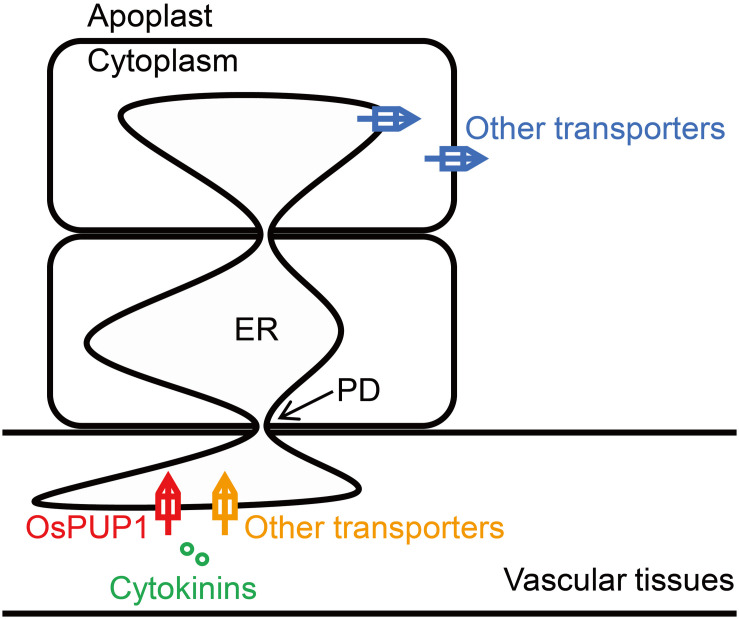
Proposed working model for the function of OsPUP1 in CK transport system. OsPUP1 is localized to endoplasmic reticulum (ER) in the cells of vascular tissues, and might functions as an influx transporter together with other CK transporters in importing CKs from cytoplasm into ER, which might facilitate the cell-to-cell movement of CK through the plasmodesmata (PD).

Plasmodesmata provide efficient channels for molecules to move from cell-to-cell via the ER lumen (Barton et al., 2011). As overexpression of *OsPUP1* resulted in phenotypes almost contrary to those of *BG3/OsPUP4*- or *OsPUP7*-overexpressing plants, especially regarding the plant height and grain weight, we hypothesized that, while BG3/OsPUP4 and OsPUP7 function in loading CK into vascular tissues (Xiao et al., 2019), OsPUP1 might be involved in unloading CK out from vascular tissues (Figure 6). The ER-localized OsPUP1 might function as an influx transporter together with other CK transporters in importing CKs from cytoplasm into ER of cells in vascular tissues. Considering the role of root-derived tZ in promoting the shoot growth (Takei et al., 2004; Gao et al., 2014; Ko et al., 2014; Zhang K. et al., 2014), the reduced content of tZ and tZR in the shoot might be the reason for the dwarfism of *OsPUP1*-overexpressing plants. It should be mentioned that *OsPUP1* was expressed much more higher in the root than other tissues in the shoot (Figure 1A), which is quite different from the expression pattern of *BG3/OsPUP4* and *OsPUP7*. The root-preferential expression of *OsPUP1* suggests the potential role in unloading shoot-derived or phloem-transported CK for root growth and development. The decreased efficiency of the root-to-shoot transport of root-applied CKs in *OsPUP1*-overexpressing plants also supported this hypothesis. As *OsPUP1* was predominantly expressed in vascular tissues, particular in phloem (Figure 1B), the gene might play a role in unloading the systemic transport of CKs to regulate shoot growth and development. Thus, the identification of OsPUP1 could represent a distinct CK transporter, whose functions differ from those of OsPUP4 and OsPUP7. Apparently, these two types of CK transporters collaborated with each other contributing to the efficient hormone transportation. Together with many other additional homologs, they may form an efficient loading and unloading system to fulfill the long transport of CK.

Cytokinin receptors are suggested to be mainly localized in ER, and the perceiving CHASE domain is supposed to be exposed to the ER lumen (Caesar et al., 2011; Lomin et al., 2011, 2018; Wulfetange et al., 2011; Hwang et al., 2012; Ding et al., 2017; Romanov et al., 2018; Kubiasová et al., 2020). Considering the subcellular localization of OsPUP1 in ER and expression in cells other than those of vascular tissues (Figures 1B,D), OsPUP1 might play a role in importing CKs from cytoplasm into ER, somewhat like the role of AtPUP14 for transporting CK from apoplast to cytoplasm (Zürcher et al., 2016), to regulate the CK pool for signal perception. If OsPUP1 imports CK into ER, overexpression of *OsPUP1* might increase the CK signal transduction. However, the signal extents reflected by the expression level of *OsRRs* were not markedly changed in the roots of *OsPUP1*-overexpressing seedling (Figure 4). One possibility is that the cells producing active CKs might not be the cells containing effective CK receptors. In this case, overexpression of *OsPUP1* restricts CKs out from the cells responsible for active CK synthesis to cells responsible for CK perception. Thus, the functions of OsPUP1 may depend on cells, tissues, actual developmental stages, as well as environmental conditions.

In rice, cZ-type CKs account for the largest proportion of CKs (Supplementary Table 1; Kudo et al., 2012; Kamada-Nobusada et al., 2013; Osugi and Sakakibara, 2015). It has been reported that cZ can induce CK-dependent responses (Figure 4; Kudo et al., 2012; Silva-Navas et al., 2019). In our study, the total content of CK nucleobases and nucleosides in the roots of *OsPUP1*-overexpressing plants were increased, mainly due to the increase of cZ and cZR (Figure 3D). However, the CK response was not markedly changed, as indicated by the expression of *OsRR* genes in the roots without CK treatment (Figure 4). In addition, the content of cZOG was significantly increased in both shoots and roots of *OsPUP1*-overexpressing seedlings, but has little change in the panicles (Supplementary Table 1). Since the physiological significance and homeostasis of cZ and its conjugated forms have not been fully elucidated so far, the reason underlying these intriguing observations remain unclear.

Although application of either of the iP, tZ or cZ in root is able to induce the CK response in the shoot, the extents are quite different, that is, the induction effect of iP is much lighter than those of tZ and cZ (Figure 4). These results imply that the root-to-shoot efficiency of the translocation of iP could be lower than those of tZ and cZ, which might result from the low recognition efficiency of iP or affinity of the responsible transporters for loading iP into the vascular tissues in the root. As the CK receptors in the shoots are suggested to be usually less sensitive to iP (Heyl et al., 2012; Lomin et al., 2012), another possibility is that CK receptors have a relatively low-affinity binding to iP in the shoots.

Overexpression of *OsPUP1* caused marked morphological alterations, while the knockout mutants showed no phenotypic change. Similar observation has been reported in our previous studies, showing that both the single and the double mutants of *OsPUP4* and *OsPUP7* are phenotypically silent (Xiao et al., 2019). Thus, there should be existed strong functional redundancy among PUP members in plant, which, on the other hand, implies the importance of the hormone transportation system (Zürcher et al., 2016). Further efforts uncovering this complicated system are significant for understanding hormone functions in plant growth and development. Given the crucial roles of the OsPUP1 as well as OsPUP4 and OsPUP7 in regulating several key agronomic traits, comprehension of the hormone transport certainly has a great potential for crop improvement as has been exemplified in a recent study (Yin et al., 2020).

## Data Availability Statement

All relevant data can be found within the manuscript and its supporting materials.

## Author Contributions

YX performed most of the experiments with the assistance of JZ, GY, XL, WM, HD, GZ, GC, and HT. YX, HT, CC, and WT designed the study, analyzed the data, and wrote the manuscript. CC and WT conceived and supervised the study. All authors contributed to the article and approved the submitted version.

## Conflict of Interest

The authors declare that the research was conducted in the absence of any commercial or financial relationships that could be construed as a potential conflict of interest.

## Footnotes

1 https://www.arabidopsis.org/

2 http://coffee-genome.org/

3 http://rice.plantbiology.msu.edu/

## Supplementary Material

Supplementary Figure 1 Effect of different kinds of phytohormones on *OsPUP1* expression in shoots and roots of the wild-type seedlings.

Supplementary Figure 2 Subcellular localization analyses of OsPUP1 in rice protoplasts.

Supplementary Figure 3 Subcellular localization analyses of OsPUP1 in tobacco epidermal cells.

Supplementary Figure 4 Comparison of the plant height, tiller number, and leaf length of wild-type and *OsPUP1*-overexpressing plants.

Supplementary Figure 5 Panicle characteristics of *OsPUP1*-overexpressing plants.

Supplementary Figure 6 Mutation information in the knockout mutants of *OsPUP1*.

Supplementary Figure 7 Phylogenetic analysis of PUP family proteins in *Arabidopsis* (At), coffee (Cc), and rice (Os).

Supplementary Figure 8 Gene expression analyses of OsPUP1 and BG3/OsPUP4.

Supplementary Table 1 Quantification of various CK forms in shoots, roots, and panicles of the wild-type (WT) and *OsPUP1*-overexpressing plants (*OE2*).

Supplementary Table 2 Primers used for analyses.
