# Supplementary material for: Endoplasmic Reticulum-Localized PURINE PERMEASE1 Regulates Plant Height and Grain Weight by Modulating Cytokinin Distribution in Rice
Source: Front Plant Sci. 2020 Dec 22;11:618560. doi: 10.3389/fpls.2020.618560 (PMC7783468; doi:10.3389/fpls.2020.618560)
Supplement: Supplementary Figure 1 — Effect of different kinds of phytohormones on OsPUP1 expression in shoots and roots of the wild-type seedlings. [file Data_Sheet_1.PDF]

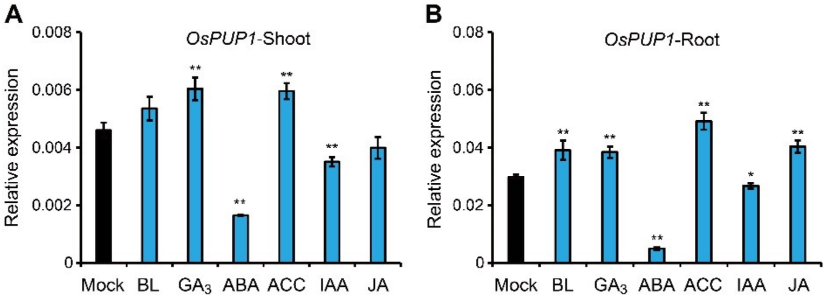

Supplementary Figure 1. Effect of different kinds of phytohormones on *OsPUP1* expression in shoots and roots of the wild-type seedlings. The roots of 8-day-old seedlings grown in 1/2 strength MS hydroponic medium were treated by different phytohormones, including brassinolide (BL), gibberellin (GA_3), abscisic acid (ABA), 1-aminocyclopropane-1-carboxylic acid (ACC), indole-3-acetic acid (IAA), and jasmonic acid (JA), at 10 μM concentrations for 4 h. After treatment, the shoot and root tissues were separately collected for gene expression analyses. *Ubiquitin2* gene was used as the internal reference. $n = 3$, bar = SD, $*P < 0.05$ and $**P < 0.01$ in Student's *t*-test.

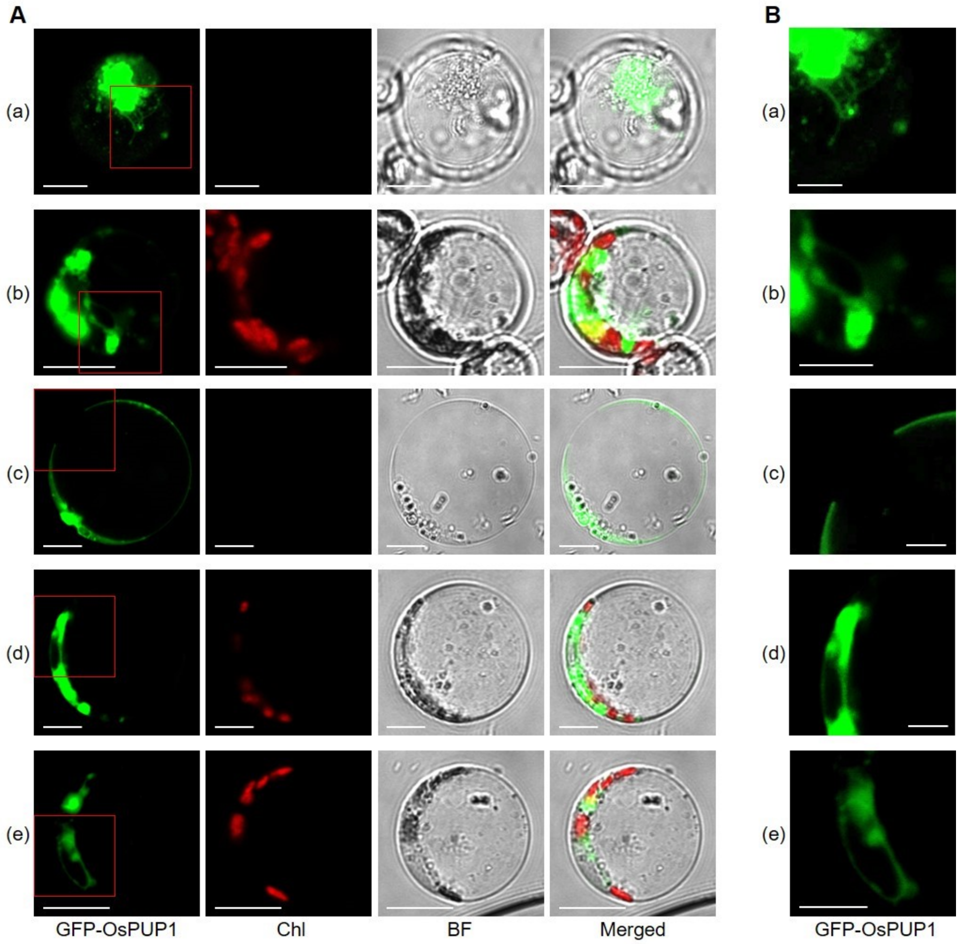

Supplementary Figure 2. Subcellular localization analyses of OsPUP1 in rice protoplasts. **(A)** Multiple cells expressing GFP-OsPUP1. **(B)** Amplification of the red-framed regions of the respective cells in **(A)**. Chl, chloroplast fluorescence; BF, bright field. Scale bars: 10 μm in **(A)**, 5 μm in **(B)**.

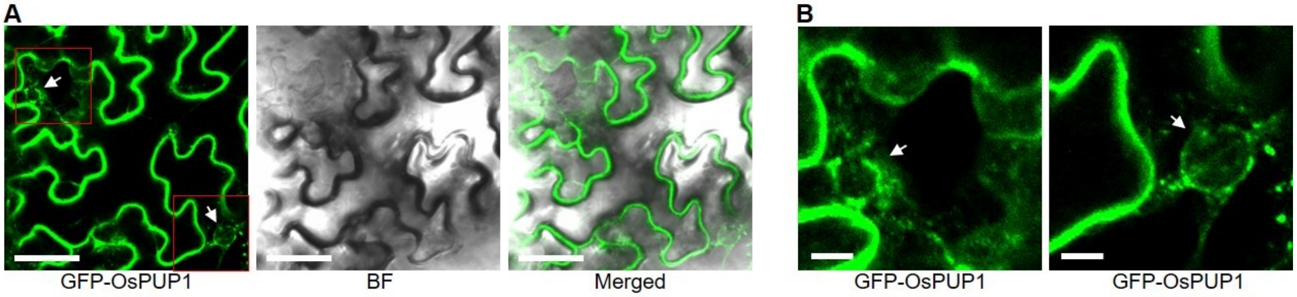

Supplementary Figure 3. Subcellular localization analyses of OsPUP1 in tobacco epidermal cells. **(A)** Multiple cells expressing GFP-OsPUP1. **(B)** Amplification of the red-framed regions of the respective cells in **(A)**. White arrowheads mark ER surrounding the nuclei. BF, bright field. Scale bars: 50 μm in **(A)**, 10 μm in **(B)**.

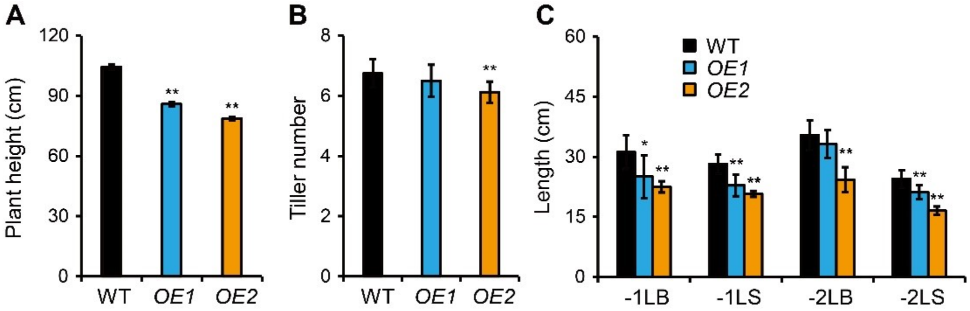

Supplementary Figure 4. Comparison of the plant height, tiller number, and leaf length of wild-type and *OsPUP1*-overexpressing plants. **(A-C)** Statistical data of the plant height **(A)**, tiller number **(B)**, and leaf length **(C)** of *OsPUP1*-overexpressing plants at mature stage, compared with the wild type (WT). n = 8, bar = SD, $*P < 0.05$ and $**P < 0.01$ in Student's t-test. -1 and -2 mean the first (also named flag leaf) and the second leaf counted from the top, respectively. LB, leaf blade; LS, leaf sheath.

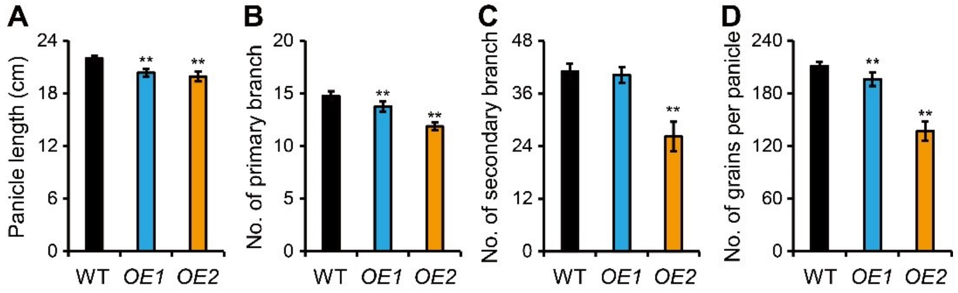

Supplementary Figure 5. Panicle characteristics of *OsPUP1*-overexpressing plants. **(A-D)** Statistical data of the panicle length **(A)**, number of primary branch **(B)**, secondary branch **(C)**, and grains per panicle **(D)** of *OsPUP1*-overexpressing plants (*OE*), compared with wild type (WT). n = 8, bar = SD, $**P < 0.01$ in Student's t-test.

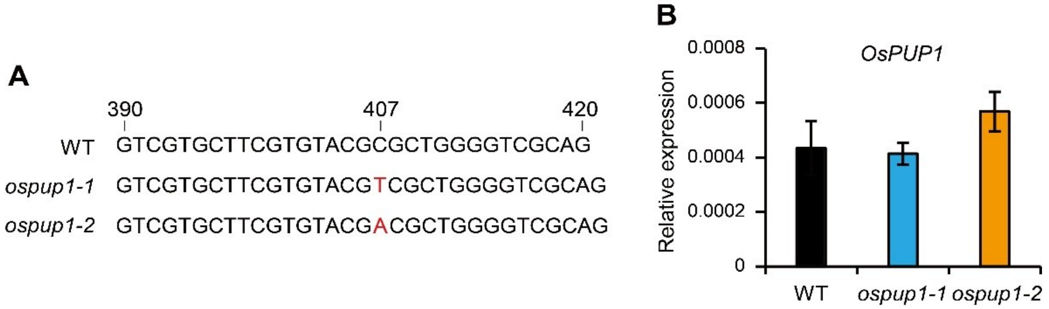

Supplementary Figure 6. Mutation information in the knockout mutants of *OsPUP1*. (**A**) The mutation sites in the coding sequence of *OsPUP1* were marked in red letter. (**B**) Relative expression of *OsPUP1* in shoots of 6-day-old overexpressing plants seedlings tested by qRT-PCR, compared with the wild type (WT). *Ubiquitin2* gene was used as the internal reference. *n* = 3, bar = SD.

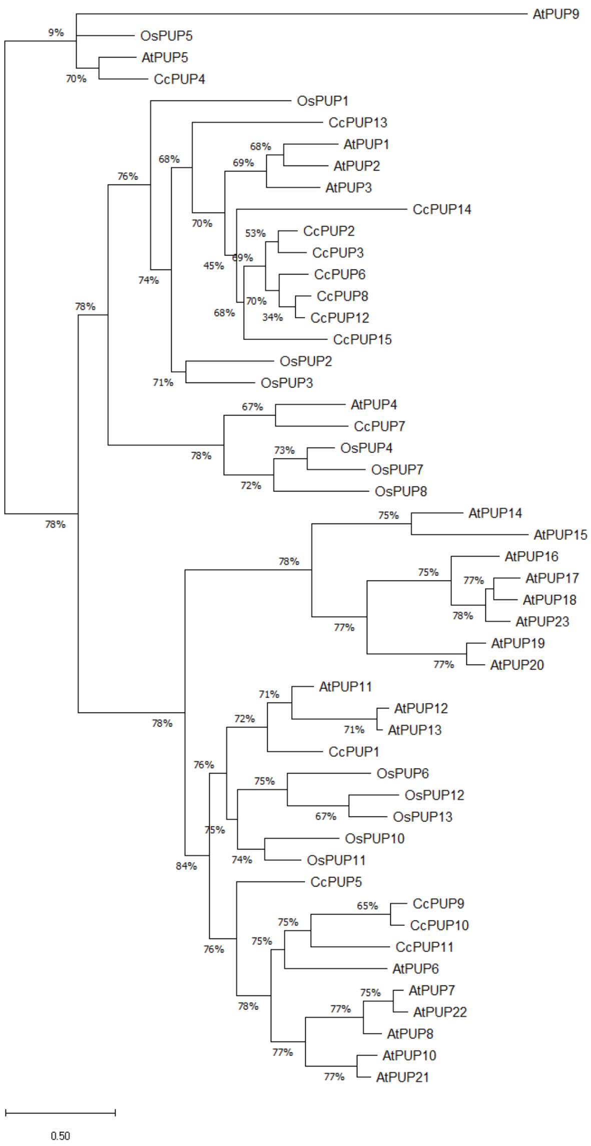

Supplementary Figure 7. Phylogenetic analysis of PUP family proteins in *Arabidopsis* (At), coffee (Cc), and rice (Os). The phylogenetic tree was constructed by software MEGA. The distance mode was observed divergence. Percentage indicated the data coverage.

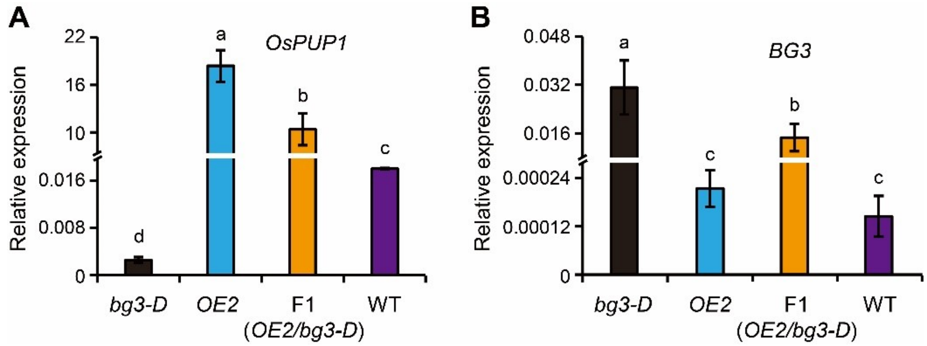

Supplementary Figure 8. Gene expression analyses of *OsPUP1* and *BG3/OsPUP4*. **(A,B)** Gene expression of *OsPUP1* **(A)** and *BG3/OsPUP4* **(B)** in the 5-day-old seedlings of *bg3-D* mutant, *OsPUP1*-overexpressing plant, their F_1 progeny, and the wild type (WT). *Ubiquitin2* gene was used as the internal reference. $n = 3$, bar = SD. Different letters above the columns indicate statistically significant differences between groups (*t* Test LSD, $P < 0.05$).

Supplementary Table 1. Quantification of various cytokinin forms in shoots, roots, and panicles of the wild-type (WT) and *OsPUP1*-overexpressing plants (*OE2*). $n = 3$, value = means ± SD (pmol·g^{-1} F.W.). FW, fresh weight. n.d., not detected. Asterisks indicate statistically significant difference compared with WT. *$P < 0.05$ and **$P < 0.01$ in Student's *t*-test.

Analyte	WT-Shoot	*OE2*-Shoot	WT-Root	*OE2*-Root	WT-Panicle	*OE2*-Panicle
iP	0.44 ± 0.00	0.30 ± 0.05**	n.d.	n.d.	1.13 ± 0.00	0.98 ± 0.00**
iPR	0.45 ± 0.05	0.37 ± 0.23	1.13 ± 0.03	0.98 ± 0.14	9.61 ± 0.17	7.69 ± 0.21**
iP7G	0.25 ± 0.03	0.26 ± 0.02	0.27 ± 0.00	0.26 ± 0.09	1.32 ± 0.06	1.74 ± 0.07**
iP9G	1.04 ± 0.07	1.32 ± 0.48	0.47 ± 0.00	0.24 ± 0.02**	2.03 ± 0.06	2.97 ± 0.06**
tZ	0.61 ± 0.03	0.36 ± 0.05**	0.14 ± 0.00	0.12 ± 0.03	0.21 ± 0.03	0.18 ± 0.00
tZR	0.23 ± 0.00	0.14 ± 0.00**	0.39 ± 0.04	0.26 ± 0.03*	0.79 ± 0.02	0.78 ± 0.02
tZ7G	n.d.	n.d.	n.d.	n.d.	n.d.	n.d.
tZ9G	3.91 ± 0.70	2.96 ± 0.33	4.04 ± 0.07	4.08 ± 0.12	2.00 ± 0.07	1.80 ± 0.08*
tZOG	n.d.	n.d.	n.d.	n.d.	n.d.	n.d.
cZ	7.28 ± 0.05	7.66 ± 0.53	2.31 ± 0.03	4.33 ± 0.00**	0.29 ± 0.03	0.33 ± 0.03
cZR	4.34 ± 0.33	5.30 ± 1.42	7.65 ± 0.14	9.46 ± 0.29**	5.76 ± 0.09	4.87 ± 0.09**
cZ9G	0.68 ± 0.12	0.59 ± 0.03	0.62 ± 0.02	0.65 ± 0.04	n.d.	n.d.
cZOG	2870.01 ± 40.98	3561.89 ± 164.04**	1931.92 ± 36.15	2661.59 ± 71.14**	430.49 ± 18.43	411.44 ± 22.00
DHZ	0.21 ± 0.03	0.15 ± 0.03*	n.d.	n.d.	0.00 ± 0.00	0.05 ± 0.00**
DHZR	0.09 ± 0.02	0.08 ± 0.05	0.09 ± 0.02	0.15 ± 0.02*	0.03 ± 0.00	0.06 ± 0.00**
DHZOG	0.23 ± 0.03	0.17 ± 0.02	0.05 ± 0.00	0.10 ± 0.03*	n.d.	n.d.

iP, *N*^{6}-($\triangle^2$-isopentenyl) adenine (or isopentenyladenine); iPR, isopentenyladenine riboside; iP7G, isopentenyladenine riboside 7-glucoside; iP9G, isopentenyladenine riboside 9-glucoside; tZ, *trans*-zeatin; tZR, *trans*-zeatin riboside; tZ7G, *trans*-zeatin 7-glucoside; tZ9G, *trans*-zeatin 9-glucoside; tZOG, *trans*-zeatin O-glucoside; cZ, *cis*-zeatin; cZR, *cis*-zeatin riboside; cZ9G, *cis*-zeatin 9-glucoside; cZOG, *cis*-zeatin O-glucoside, DHZ, dihydrozeatin; DHZR, dihydrozeatin riboside; DHZOG, dihydrozeatin O-glucoside.

Supplementary Table 2. Primers used for analyses. Additional bases used for plasmid construction are underlined.

Name	Forward (5'-3')	Reverse (5'-3')
**For vector construction:**		
OsPUP1-OE	CCGGGGATCCTCTAGAATGGCCACCATTACTGCT	AAAGCAGGGCATGCCTGCAGCTAAGGCGCCGCTGACTC
pOsPUP1::GUS	TACGCCAAGCTTGGCTGCAGCTTCTAGGCTTCTAGCACT	GAATTCCCGGGGATCCGATCAAATGAAGCAGTGCT
GFP-OsPUP1	CAAGGAGCTCGGATCCATGGCCACCATTACTGCT	GCAGGTCGACTCTAGACTAAGGCGCCGCTGACTC
**For qRT-PCR:**		
OsPUP1	TCGCCGTCATCTTCCTCCA	CGTCTGCGGTCTTCTGTG
Ubiquitin2	GAGCCTCTGTTCGTCAAGTA	ACTCGATGGTCCATTAAACC
BG3/OsPUP4	ATGGTGTACCTCACGTCGT	TCTTCTGCGTGGTATACT
OsRR1	AGGATCAGCAGATGCATGAATG	GAGACGCTGTACGTCCTTGCTT
OsRR2	CATGGTGATGAATGCATCC	TGCTGCCATTGGACCATCT
OsRR4	TGAAGCTGCAACAGCTCA	AGTGGAGGACAATCTTGG
